# Diuretic Resistance in Heart Failure

**DOI:** 10.1007/s11897-019-0424-1

**Published:** 2019-04

**Authors:** Richa Gupta, Jeffrey Testani, Sean Collins

**Affiliations:** 1 Department of Cardiovascular Medicine, Vanderbilt University Medical Center, 1121 Medical Center Dr., Nashville, TN 37212; 2 Department of Emergency Medicine, Vanderbilt University Medical Center, 1313 21st Ave. S, 703 Oxford House, Nashville, TN 37232; 3 Department of Cardiovascular Medicine, Yale Medical Center, PO Box 208017, New Haven, CT 06520

**Keywords:** Acute heart failure, Loop diuretics, Diuretic resistance, Spot urine sodium, Biomarkers

## Abstract

**Purpose of review:**

Diuretic resistance (DR) occurs along a spectrum of relative severity and contributes to worsening of acute heart failure (AHF) during an inpatient stay. This review gives an overview of mechanisms of DR with a focus on loop diuretics and summarizes the current literature regarding the prognostic value of diuretic efficiency and predictors of natriuretic response in AHF.

**Recent findings:**

The pharmacokinetics of diuretics are impaired in chronic heart failure, but little is known about mechanisms of DR in AHF. Almost all diuresis after administration of a loop diuretic dose occurs in the first few hours after administration and within-dose DR can develop. Recent studies suggest that DR at the level of the nephron may be more important than defects in diuretic delivery to the tubule. Because loop diuretics induce natriuresis, urine sodium (UNa) concentration may serve as a functional, physiologic and direct measure for diuretic responsiveness to a given loop diuretic dose.

**Summary:**

Identifying and targeting individuals with DR for more aggressive, tailored therapy represents an important opportunity to improve outcomes. A better understanding of the mechanistic underpinnings of DR in AHF is needed to identify additional biomarkers and guide future trials and therapies.

## Introduction

The prevalence of hypertension, chronic heart failure (HF), and chronic kidney disease are increasing, and cardiovascular disease is currently the most common cause of death and disability worldwide [1]. HF affects nearly 6 million people in the United States. In over one million annual hospital discharges, acute heart failure (AHF) is the primary diagnosis [2]. Development of congestion leading to AHF decompensation powerfully predicts poor patient outcome [3, 4]. Despite symptom improvement, at least 50% of patients experience no weight loss [5] and up to 50% leave the hospital with residual congestion which predicts additional readmissions and higher mortality [3, 6–9].

Diuretic resistance (DR) has no single accepted definition, but the most frequently cited is “failure to decongest despite adequate and escalating doses of diuretics” (10, 11). DR is a relative term, as, there exists a broad range of diuretic *efficiency* (DE), defined as the efficiency with which a diuretic can facilitate diuresis and natriuresis, rather than the absolute dose of diuretic [12]. Relatively few patients are completely diuretic “non-responsive,” however, depending on the bar set, 20–50% of hospitalized patients have a poor initial response to IV loop diuretics and are deemed “diuretic resistant [13].” While normal response to a diuretic has been defined as 3–4 L per 40 mg of furosemide, diuretic resistant individuals exhibit impaired DE along a spectrum of relative severity [11, 13]. DR in patients has been shown to contribute to worsening heart failure (WHF) during inpatient stay, prolonged lengths of stay, and likely increased mortality and the consumption of more resources relative to those who respond adequately to initial diuretic administration [14–16]. Identifying and targeting these individuals for more aggressive, tailored therapy thus represents an important opportunity to improve outcomes.

Diuretics are the cornerstone of AHF therapy. The therapeutic effect of diuretics is a function of loss of body sodium and fluid [17, 18]. Most AHF admissions are due to volume overload and treated with intravenous (IV) loop diuretics. There is, however, currently no consensus on adjustment of IV loop diuretic doses based on individual responses to initial diuretic. In fact, because diuretic dosing and response vary widely, many patients are left inadequately treated [7]. To reduce the prevalence of WHF and optimize treatment delivery, the individual response to diuretics must be predictable so DR can be readily recognized, and therapy can be intensified.

The scope of this review focuses specifically on the characteristics of loop diuretics. First, we will outline the mechanism and pharmacokinetics of these drugs, showing the majority of natriuresis occurs shortly after the administration of a loop diuretic dose. Next, we discuss the diuretic “braking” phenomenon and mechanisms of DR in AHF, highlighting the importance of intra-renal DR. We then discuss the concept of loop DE as it relates to quantifying diuretic responsiveness, describing the literature to date that has focused on early-response prediction, highlighting the use of urine sodium (UNa) as an objective measure of natriuresis following loop diuretic administration. Finally, we will review the limitations of current approaches to AHF management and raise important remaining questions which, if answered, may guide future direction in reducing congestion, readmission rates and cardiovascular mortality.

## Mechanism of loop diuretics

**Fig 1.**
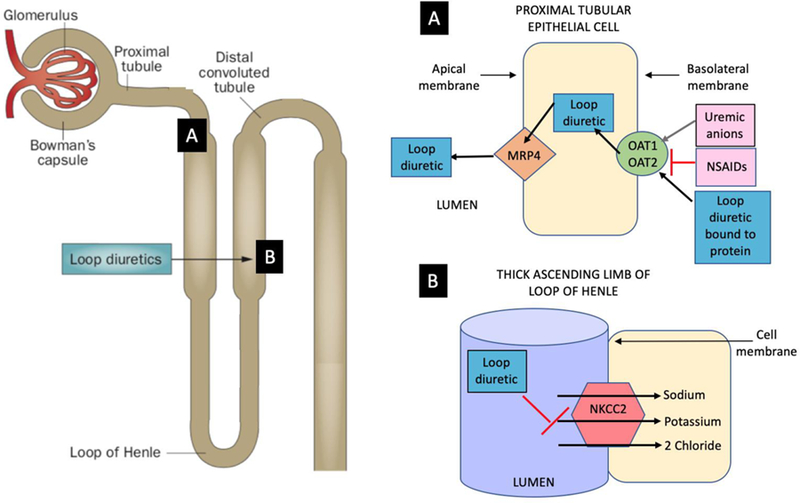
Diuretic secretion by the proximal tubule and diuretic action on the Loop of Henle. A. Proximal convoluted tubule: After translocation into the proximal tubule cell, the loop diuretic is then secreted across the basolateral or luminal membrane by voltage-driven organic anion transporters (OAT1 and OAT2) and at the apical membrane by multidrug resistance-associated protein 4 (MRP4) and others. B. Thick ascending limb of the loop of Henle: The primary action of loop diuretics occurs here on the luminal membrane where an electroneutral Na-K-2Cl (NKCC2) is located. This cotransporter mediates sodium and chloride movement across the apical membrane. Loop diuretics bind to the NKCC2 from the luminal surface to block the reabsorption of sodium and chloride across the apical membrane via this transporter. The tubular lumen becomes more hypertonic and the interstitium less so, diminishing the osmotic gradient required for water reabsorption.

The first opportunity for an adequate diuretic response hinges on pharmacokinetics or the effective delivery of loop diuretics to inhibit the Na-K-2Cl cotransporter (NKCC2), as shown in Figure 1, and subsequently on their ability to produce a natriuresis (pharmacodynamics). Diuretics are first absorbed into the bloodstream via the gut, and the kinetics of absorption of loop diuretics varies after ingestion [19, 20]. Oral bumetanide and torsemide are absorbed more completely and reproducibly than furosemide. Furosemide has been reported to have bioavailability ranging from 10–90% (average 50%), and a doubling of the furosemide dose is considered equivalent when changing from IV to oral. Furosemide absorption can vary over time, within and in between patients [18–20]. Food, gastric pH, gut perfusion and edema are all known or thought to influence absorption kinetics. Diuretics gain access to tubular fluid by active proximal secretion via organic anion transporters (OATs), a process dependent on renal blood flow and shown in Figure 1 [21]. Due to the high degree of protein binding there is minimal entry of loop diuretics into the tubular fluid by glomerular filtration, but rather the majority occurs via secretion by OATs [22].

## Pharmacokinetics and pharmacodynamics of loop diuretics in disease states

Table 1 summarizes the commonly cited mechanisms of DR in the literature, although these have not been specifically investigated in AHF. Moreover, the relative importance of each of these mechanisms listed remains unclear; some mechanisms, such as renal tubular defects, are likely more important than others. Acidosis and hypoalbuminemia can exert an influence on drug delivery or pharmacokinetics, while, NSAIDs, reduced dietary salt intake and repeat administration of furosemide all diminish the renal tubular response to furosemide or pharmacodynamics [23]. Metabolic acidosis can depolarize the membrane potential of proximal tubule cells [24]. This has been hypothesized to decrease organic anion (OA) secretion, thus increasing plasma levels of OA and urate and impairing proximal tubule secretion of diuretics, which in turn decreases their delivery to the active sites in the nephron (18). Albumin potentiates proximal secretion of active furosemide and thus hypoalbuminemia may impair the uptake and secretion of active furosemide and enhance conversion to the drug’s inactive form [25, 26]. These mechanisms of enhanced furosemide metabolism and decreased tubular secretion of active diuretic, however, have never been studied in patients with AHF. In general, the pharmacokinetic effects of acidosis and albumin are relatively small and may be an irrelevant source of DR [27, 28].

Chronic kidney disease (CKD) and liver disease can influence drug delivery to the nephron or affect the degree to which the drug is potentiated [18]. Furosemide is metabolized to inactive glucuronide in the kidney, while bumetanide and torsemide are metabolized in the liver only [29]. This implies that in CKD, the relative potency of furosemide increases, enhancing natriuresis. Renal clearance of loop diuretics falls in parallel with glomerular filtration rate (GFR) due to decreased renal mass (reducing perfusion or renal blood flow and thus drug delivery) and accumulation of OAs such as blood urea nitrogen, which compete with proximal secretion of diuretic via OATs, further reducing diuretic availability at the site of action [16, 21]. The maximal increase in fractional excretion of sodium produced by furosemide can be maintained well in CKD with a higher furosemide dose. The absolute response to diuretics, however, is still limited by the loss of nephrons which reduces the amount of absolute diuresis, even if each nephron is maximally excreting sodium. This is because renal blood flow is proportional to nephron mass [30, 31].

**Fig 2.**
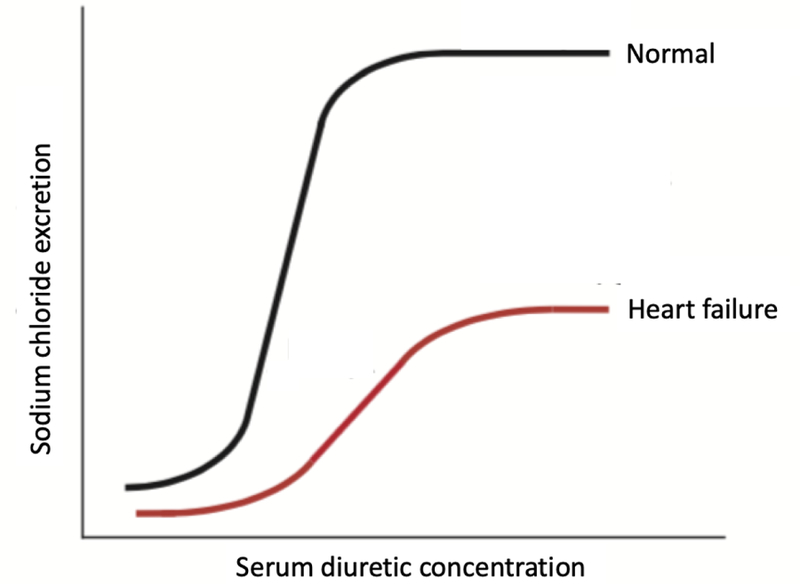
Dose-response curve for loop diuretics with sodium chloride excretion as a function of plasma loop diuretic concentration. Note a rightward shift in the curve due to diuretic resistance in a patient with chronic heart failure compared to normal subjects. In heart failure, large increases in diuretic dose are required to achieve modest increases in sodium chloride excretion.

In *chronic* HF, the absorption kinetics of diuretics are impaired and these individuals, relative to people without HF, are diuretic resistant [20, 27, 32]. In HF, the loop diuretic dose-response curve is shifted down and to the right with attenuated response compared to normal subjects [18, 33]. This log-linear relationship, illustrated in Figure 2, implies that it takes large increases in the dose of loop diuretic to achieve modest increases in diuresis. This relationship, however, has thus far not been studied in AHF. Recent literature suggests that defects at the level of the renal tubule are substantially more important than reduced diuretic delivery in determining DR in patients with AHF [34]. The authors of this study demonstrate two important points: 1) the amount of administered loop diuretic that actually reaches the tubule is highly variable between individual AHF patients and 2) urine diuretic concentration and delivery to the tubule is linearly related to serum concentration and the IV dose of diuretic. This suggests massive defects in drug secretion would be necessary to meaningfully alter DR. Thus, defects in drug delivery explain only a minor proportion of DR in an AHF population, implying that renal tubular defects are more important. Finally, AHF results from an imbalance in neurohormonal systems that regulate cardiac and renal function. Renin-angiotensin-aldosterone system (RAAS) and sympathetic nervous system (SNS) activation are traditionally implicated in the literature as mechanisms of diuretic resistance [11]. Through a myriad of mechanisms that evolved for the control of sodium and water balance, neurohormonal activation increases sodium retention, largely through direct effects on solute transport in various nephron segments [35].

It is for these reasons that higher doses of loop diuretic are often required to achieve maximal natriuretic response, which is generally substantially less than can be achieved in health or in CKD [36]. A key point to therapy in AHF is that while aggressive decongestion is associated with worsening renal function (WRF), survival appears to be paradoxically improved [37] The development of WRF, in some cases, suggests a high degree of decongestion is occurring, and those with preserved or improved renal function may be underdiuresed and leave the hospital with residual congestion. One post-hoc analysis showed that residual congestion at the time of WRF is prognostically important; patients with AHF who have WRF (creatinine increase of ≥ 0.3 mg/dL) were shown to have longer average length of hospital stay and a greater risk of death or readmission for a cardiovascular or renal reason within 30 days of assessment if they exhibited significant congestion [38]. This suggests that patients that are incompletely decongested and develop WRF are overall a sicker, higher risk group.

Lastly, pharmacogenetics may play a role in diuretic responsiveness. Studies have demonstrated certain polymorphisms can determine an individual’s response to diuretics. Vormfelde et al, for instance, demonstrated that in 97 healthy white patients, diuretics exerted greater acute effect in subjects with polymorphisms in genes encoding the sodium-chloride cotransporter (NCC) and the beta subunit of ENaC (epithelial sodium channel transporters that are distal to NKCC2), but diminished effect in those with a polymorphism in the gamma-subunit of ENaC [39]. Another study identified female gender and polymorphisms in the gene encoding one of the organic anion transporters (OATP1B1) as a predictor of slower elimination of torsemide [40]. Finally, a more recent meta-analysis of the DOSE, CARESS and ROSE trials specifically studied patients with AHF to evaluate the impact of genetic variations in renal sodium reuptake transporters on the efficacy of furosemide therapy [41]. This study, however, was largely negative and found that none of the 6 primary variants in these genes were significantly associated with net fluid loss. Further investigation in this area is needed.

## Bioavailability of loop diuretics

In disease states such as AHF, torsemide’s increased oral bioavailability may make it a favorable drug of choice in the outpatient setting [20, 32, 33]. In AHF, furosemide is erratically bioavailable compared with bumetanide or torsemide. Recent literature suggests these differences between the drugs have clinical implications. One open label study found a 50% reduction in readmission to the hospital in patients with HF who were randomly assigned to receive torsemide rather than furosemide [42]. Another estimated that treatment with torsemide is more cost-effective than therapy with furosemide [43]. Long-term outcomes using torsemide compared with furosemide are currently being further studied in TRANSFORM (NCT03296813), a large-scale, randomized clinical effectiveness study of 6,000 patients recently hospitalized with HF.

## Duration of action of loop diuretics

The duration of action of loop diuretics after oral dosing is short (generally only 2 to 4 hours), even when pharmacologic doses of drug remain in the tubular fluid. This results in abrupt, short-lived natriuresis, as DR at the level of the nephron, rather than pharmacokinetics, dictates the duration of action [44]. Notably, in a recent study with extended-release torsemide, severe within-dose development of DR was actually demonstrated [33].

Furthermore, multiple studies have demonstrated there are minimal to no differences between continuous and bolus dosing of furosemide despite the difference in pharmacokinetics between these two methods of delivery [45]. Notably, in the largest trial to date of infusion versus bolus (the DOSE trial), patients with the highest pre-admission diuretic dose (i.e., the patients with DR) had significantly lower diuresis with infusion compared to bolus [6, 46]. These observations are notable in that there are profound pharmacokinetic advantages of continuous infusion of diuretic that are somehow being defeated by rapid development of DR. The constellation of observations reinforces the idea that interventions that target purely the pharmacokinetics of loop diuretics are unlikely to be fully successful if the response of the loop diuretic at the level of the kidney is ignored.

## Loop diuretic efficiency

A growing body of literature suggests metrics of diuretic responsiveness can provide prognostic information beyond that provided by changes in weight, fluid balance or loop diuretic dose alone [12, 13]. In this section, we further discuss the concept of loop DE and outline its ability to capture prognostic information.

Loop DE was originally defined as the net fluid loss per milligram of loop diuretic equivalent administered during AHF hospitalization [12]. Several studies have shown that DE can capture distinct prognostic information beyond raw fluid output and diuretic dose. In 2014, an analysis of a combined cohort of 1047 patients suggested DE was independently associated with survival after adjusting for baseline characteristics, diuretic dose and fluid output [12]. Diuretic dose did not retain independent prognostic information in this study and low DE had an equal if not worse prognosis in patients receiving lower doses of loop diuretics, arguing against the association between in-hospital high-dose loop diuretics and mortality being cause and effect. The authors additionally found no association between GFR and DE, suggesting renal function and drug delivery (or pharmacokinetics) does not drive DE in AHF, but rather it is the impact of cardiac function, renal function and volume status on the pharmacodynamics of loop diuretics (as previously described) that is more important.

The prognostic value of DE was also demonstrated using the CARESS, ROSE and DOSE-HF trial data and outcomes [47]. In this study, DE was defined as total 72-hour fluid output per total loop diuretic dose, expressed as 40 mg furosemide equivalents; survival was compared between patients with DE above versus below the median DE. Poor diuretic response was associated with low blood pressure, diabetes, long-term diuretic use, WRF and blood urea nitrogen, poorer New York Heart Association class and fewer signs of congestion. Lower DE was a marker of HF disease severity and was associated with reduced survival in this study as well.

## Diuretic braking and implications for AHF therapy

“Diuretic braking” is a descriptive term for a reduction in diuretic response with repeated dosing. This is an observation rather than a mechanism, and the actual mechanistic underpinnings of this phenomenon, in health and disease, are poorly understood. Reduced response to diuretic has not been shown to have a clear, consistent association with detectable changes in plasma volume or renal hemodynamics, nor with class of diuretic [27].

The blunted natriuretic response to furosemide during repeated administration is attributed in the DR literature to several factors. Candidate mechanisms include reduced sodium chloride (NaCl) delivery to the site of furosemide action, resulting in decreased inhibition of NaCl reabsorption by furosemide in the loop of Henle. It is unclear, however, if there is significant relevance to these mechanisms in humans. One study evaluated 128 patients with AHF receiving treatment with loop diuretics. The authors demonstrated that endogenous lithium clearance (a surrogate for proximal tubular sodium reabsorption) in AHF patients was no different compared to controls; most patients had a robust increase in lithium clearance following loop diuretic administration, indicating preserved sodium reabsorption response at the loop of Henle [28]. Another potential mechanism of DR has been shown in studies of rats receiving very high sodium diets. In these rats, several days of loop diuretic infusion caused structural hypertrophy of the distal convoluted tubule, connecting tubule and intercalated cells of the collecting duct [48]. These structural and functional adaptations result in an increase in the Na-K-ATPase activity and NCC expression in these downstream nephron segments, compensating for increased sodium exit from the loop of Henle induced by loop diuretics [49]. This leads to enhanced distal NaCl absorption, leading to inappropriate renal sodium retention in these animals that can persist for up to 2 weeks after cessation of diuretic therapy [49]. Rao et al, in the same study discussed above, showed some translation of this mechanism to humans. Again, by studying the fractional excretion of Lithium to assess proximal tubular and loop of Henle sodium handling, the authors showed that distal compensatory sodium reabsorption makes the largest relative contribution to diuretic-induced increase in the fractional excretion of sodium (FENa), and thus is a primary driver of DR [28].

These mechanisms of DR leave substantial uncertainty in clinical implications and recommendations for therapy in AHF. For instance, salt restriction to create a negative sodium chloride balance and compensate for post-diuretic salt retention is often a mainstay of inpatient therapy for AHF, even in the setting of receiving powerful loop diuretics [50]. Contemporary literature, however, would suggest that in AHF this may not be true; restriction of salt intake can be difficult in clinical practice and has been suggested to not be associated with improved outcomes [51]. A second mainstay of therapy during prolonged diuretic administration is to administer sequential nephron blockade by using additional classes of diuretic (for instance thiazides or carbonic anhydrase inhibitors) to overcome resistance and prevent post-diuretic sodium retention after cessation of loop diuretic activity. To date, however, studies to evaluate combination therapy in patients with AHF who have DR are scarce and inconclusive [11]. Some observational literature has actually suggested potential harm of early addition of thiazide diuretics [52]. However, a recently funded study should provide additional information about the combination of loop diuretics and carbonic anhydrase inhibitors [53]. Third, diuretics with more prolonged action or more frequent administration have been previously hypothesized to enhance NaCl loss by limiting time available for post-diuretic salt retention (for instance a continuous infusion). Again, this has never been objectively shown in evidence-based trial literature thus far. Lastly, there is no data to support that preventing or treating chloride-depletion alkalosis can enhance DE even though this has been proposed [54, 55]. This idea, however, has not actually been tested.

## Measures of diuretic resistance

In hospitalized patients, accurately accounting for actual fluid intake and elimination in AHF patients using measured output and/or standing weights can be labor-intensive, inaccurate and impractical. Real-time, objective measures of DE and natriuretic response may result in more individualized diuretic dosing to maximize AHF treatment. Over-activation of the RAAS/SNS activity in AHF stimulates sodium absorption. UNa, is thus a functional, physiologic, measurement that may represent a product of this activation in AHF. Because loop diuretics induce natriuresis, UNa concentration may serve as a direct measure for diuretic responsiveness that integrates multiple sources of DR and better reflects the response to a given loop diuretic dose. The inability to relieve congestion as predicted by sodium output may thus indicate more advanced disease.

A growing body of literature suggests that as an integrative measure of diuretic resistance, UNa can predict short term responsiveness to IV loop diuretics in patients with AHF. To date, several studies (summarized in Table 2) have been published that support the utility and value of UNa measurement. In 2014, a study of 52 patients with AHF suggested high spot UNa concentration assessed at steady state during continuous infusion of furosemide was associated with volume of urine output (UOP) and fewer adverse events [56]. This study suggests a strong correlation between UNa and DE in the setting of loop diuretic use. In a subsequent study, UNa > 60 mmol/L measured at day 3 of a hospitalization for AHF was found to be associated with improved 180-day outcomes of AHF hospitalization and cardiovascular mortality [57]. Finally, a recently published study found that patients hospitalized with AHF with low spot UNa concentration (≤ 60 mmol/L) after first IV diuretic administration are at increased risk for incomplete decongestion and adverse events compared to patients with intact natriuresis [58].

Further investigations (Table 2) have focused on practical methods to rapidly predict diuretic response. A prospective study of 50 patients with AHF used meticulously-collected 6-hour urine collections to quantify cumulative sodium output and derive an equation to predict net sodium output using a spot urine sample obtained 1 or 2 hours after loop diuretic administration [59]. The study found an excellent correlation between cumulative UNa output and predicted UNa output at 6 hours with application of this equation to UNa measurements collected 1 and 2 hours after IV diuretic administration. Other parameters such as sodium output, fluid output, weight change, UNa concentration, fractional excretion of sodium, and diuretic dose all had weaker correlations than that predicted from the equation. Thus, poor natriuretic response (defined as a cumulative sodium output < 50mmol) could be accurately predicted with this equation. More recently, a study of 176 patients with AHF receiving IV furosemide investigated whether a single spot UNa measured at first void and at 3 hours in the outpatient setting could predict initial response [60]. The study suggested higher UNa predicted UOP after adjusting for age, BUN and GFR. It was also associated with a lower risk of hospitalization or ED visit in the subsequent 30 days even after adjusting for systolic blood pressure, hemoglobin and urine volume. The authors further identified cutoffs of a spot UNa in first voided urine after diuretic administration that was greater than 65 mmol and UOP greater than 1.2 L to be associated with a significantly lower risk of 30-day hospitalization with this algorithm. Finally, a prospective study of the value of spot urine electrolytes 1 hour after first dose of diuretic upon ED presentation was conducted in 61 patients [61]. Urine electrolytes were measured and patients were followed during their hospitalization for WHF, defined as persistent or worsening AHF symptoms requiring intensification of AHF therapy during the first five days of hospitalization. The investigators showed that UNa at one hour < 35.4 mEq/L was 100% sensitive for predicting WHF and those with WHF had lower UNa and UOP at 1 hour and a longer inpatient length of stay.

In summary, spot UNa concentration can add information beyond empiric response to diuretic infusion or other clinical variables. UNa is a marker of natriuresis that is easily measurable, readily available and reliably prognostic. Further study of urine chloride is also warranted for similar reasons.

## Future Directions

The aforementioned prospective studies suggest spot UNa is a readily available measure of natriuresis and could be used to guide AHF therapy [62]. However, randomized trials are needed to determine the incremental benefit of this approach relative to standard clinical management. Furthermore, additional investigation of biomarkers and molecular determinants of diuretic response beyond UNa are needed to determine their role in predicting HF, HF readmission and cardiovascular death; little to no investigation exists in this area. These studies, however, might lay the foundation for further investigations evaluating alternative diuretic strategies and their impact on ENaC expression and DE. Lastly, more accurate measures of decongestion, such as the clinical congestion score, should be utilized in the methodology of future trials to quantify the impact of AHF therapy during hospitalization and at the time of discharge [3]. Other objective measures of decongestion need to be further explored as we determine the optimal method of decongestion. Studies of changes in blood volume, for instance, suggest this may be another objective measure of decongestion [63].

## Conclusions and recommendations

Our review of DR in AHF lends itself to a few points that highlight key clinical implications of AHF management. First, almost all diuresis after administration of a loop diuretic dose occurs in the first few hours after administration and there is even within-dose DR that develops. In disease states like CKD and AHF, this resistance occurs as a direct result of the disease. Second, identifying patients anticipated to have low DE on presentation to the hospital or in the ED can facilitate triage of patients less likely to be responsive to therapy, shorten time to first diuretic dose, and guide rapid escalation of diuretic strategies. Identification of these poor-responders can also allow for their selective enrollment in RCTs for new therapies. Finally, evidence-based guideline recommendations for AHF therapy in the hospital setting are currently lacking and there is extensive interest in determining the optimal approach to initial therapy. While response to AHF therapy remains poorly defined, it is likely that greater decongestion improves outcomes.

We suggest measures of UNa may be a tool that can help optimize diuretic dosing and efficiency. Sodium and water retention due to inadequate natriuresis and diuresis are hallmarks of AHF. Poor gut absorption of orally administered loop diuretics, decreased diuretic delivery to the site of action and renal tubular compensatory hypertrophy all lead to a diminished effect of diuretics, resulting in impaired UNa excretion and the diuretic braking phenomenon, thus contributing to resistance in patients with AHF. UNa is an objective measure of natriuresis that can be measured within an hour or two of loop diuretic therapy; assays of biomarker activity show similar promise. This supports the design of a randomized controlled trial evaluating the use of these objective measures of spot UNa-directed and/or biomarker-directed diuresis to improve clinical symptoms and decrease WHF, post-discharge AHF readmission and cardiovascular mortality. Such a study would inform clinical practice and could lead to a protocolized diuretic regimen based on these dynamic changes to quickly optimize decongestion. Finally, UNa and genetic or molecular biomarkers are just examples of potential measurable tools that may guide diuretic therapy, however in reality, little is known about mechanisms of DR. Recent literature presented in this review suggests that much of this resistance occurs at the level of the nephron, which must not be ignored. Ultimately, a better understanding of the mechanistic underpinnings of DR in AHF is needed to inform the design of future trials evaluating AHF therapy.

## Figures and Tables

**Table 1:** Proposed potential causes of diuretic resistance (DR) ^†^

xPRE-NEPHRON DR	Poor absorption of diuretics due to gut edema
	Poor renal blood flow due to detrimental hemodynamic effects of other conditions such as heart failure or cirrhosis
	Hypoalbuminemia
	Competition for diuretic entry into the nephron by other organic anions/acids
INTRA-RENAL DR	Poor renal blood flow due to nephron loss
	Neurohormonal activation
	Loop diuretic dose too low or too infrequent, particularly in the setting of decreased glomerular filtration rate
	Nephrotoxic or anti-natriuretic drugs (nonsteroidal inflammatory agents, probenecid, etc.)
	Defects at the level of the renal tubule
	Distal tubular remodeling from prolonged diuretic exposure
	Pharmacogenetics—i.e. epithelial sodium channel (ENaC) transporter subtype, etc. and variable expression

† Note: The relative effects of each of these mechanisms listed remains unclear; some mechanisms, such as renal tubular defects, are likely more important than others.

**Table 2:** Studies to date that have investigated urinary sodium (U_Na_) measurements as a predictor of clinical outcomes and natriuretic responsiveness

Study	Time point of measurement of urine sample	N	Measurements	Predictive value/outcome
Singh, et al 2014	Spot sample at steady state during continuous loop diuretic infusion	52	U_Na_ < 50 mmol	Less weight loss and decreased net fluid output over 24 hours
			U_Na_: urine furosemide ratio < 2 mmol/mg	Above, and worse clinical outcomes
Ferreira, et al 2016	Spot sample on day 3 of therapy with loop diuretic +/− spironolactone	100	U_Na_ > 60 mmol/L and U_Na_: urine potassium > 2	Fewer adverse clinical outcomes
Testani, et al 2016	1–2 hours after loop diuretic administration	50	U_Na_ < 60 mmol/L cumulative in 6 hours by equation	Worse cumulative 6-hour sodium output/poor natriuretic response
Luk, et al 2018	1^st^ urine void after loop diuretic administration	103	U_Na_ < 60 mmol/L	More adverse clinical outcomes
Brinkley, et al 2018	1^st^ urine void after loop diuretic administration	176	U_Na_ < 60 mmol/L	Greater rates of 30-day hospitalization or emergency room visit
Collins, et al 2018	1 hour after loop diuretic administration	61	U_Na_ < 35 mEq/L	Worsening heart failure
